# Transformative Approaches for Sustainable Weed Management: The Power of Gene Drive and CRISPR-Cas9

**DOI:** 10.3390/genes14122176

**Published:** 2023-12-04

**Authors:** Yaiphabi Kumam, Harold N Trick, P.V. Vara Prasad, Mithila Jugulam

**Affiliations:** 1 Department of Agronomy, Kansas State University, Manhattan, KS 66506, USA; yaiphabi@ksu.edu (Y.K.); vara@ksu.edu (P.V.V.P.); 2 Department of Plant Pathology, Kansas State University, Manhattan, KS 66506, USA; hnt@ksu.edu

**Keywords:** gene drive, CRISPR-Cas9, weed management

## Abstract

Weeds can negatively impact crop yields and the ecosystem’s health. While many weed management strategies have been developed and deployed, there is a greater need for the development of sustainable methods for employing integrated weed management. Gene drive systems can be used as one of the approaches to suppress the aggressive growth and reproductive behavior of weeds, although their efficacy is yet to be tested. Their popularity in insect pest management has increased, however, with the advent of CRISPR-Cas9 technology, which provides specificity and precision in editing the target gene. This review focuses on the different types of gene drive systems, including the use of CRISPR-Cas9-based systems and their success stories in pest management, while also exploring their possible applications in weed species. Factors that govern the success of a gene drive system in weeds, including the mode of reproduction, the availability of weed genome databases, and well-established transformation protocols are also discussed. Importantly, the risks associated with the release of weed populations with gene drive-bearing alleles into wild populations are also examined, along with the importance of addressing ecological consequences and ethical concerns.

## 1. Introduction

Weeds are one of the important biotic pests responsible for crop yield losses. If uncontrolled, they can cause crop yield losses of up to 34% in major food and cash crops globally [1]. The pursuit of intensive, selective, and effective weed management practices has been a major challenge in crop production. Herbicides have remained the most popular control measure since the 1940s, owing to their effortless practicality in weed management [2]. However, over the last eight decades, their incessant use has resulted in natural selection, and consequently, the evolution of herbicide resistance in numerous agriculturally important weed species [3]. Currently, a total of 269 weed biotypes (154 dicots and 115 monocots) have been reported to be resistant to 21 of the 31 known herbicide sites of action to date [3]. Herbicide resistance in weeds is a serious threat to sustainable agricultural production across the globe.

Sustainability and ecologically friendly solutions for weed management and improved crop production are highly warranted. To achieve this, stringent agricultural practices must be adopted, augmented with improved tillage operations [4], bioherbicides [5], allelopathy [6,7], and the use of genetic pest management practices [8]. Bioherbicides and allelopathy function like herbicides by releasing toxins that interfere with the normal growth and metabolism of plants [5,7,9]. Their popularity and implementation on a large scale are limited by their inconsistency under field conditions [5], technical feasibility including long-term storage [10], lack of broad-spectrum properties [11], and lethality to non-target organisms [12]. These ultimately contribute to their low demand in the pesticide market. There is a need for viable long-term weed control options such as the use of genetic pest management (GPM) techniques for sustainable crop production.

GPM techniques leverage the principles of evolutionary biology in deciphering the inheritance pattern in sexually reproducing pest species to suppress or replace pest populations [8,13,14]. GPM by employing the ‘artificial gene drive’ technique for weed control has been theoretically proposed [15,16], albeit supporting empirical data are currently unavailable. Gene drive, also known as ‘selfish gene’, defies Mendelian genetics to allow its biased inheritance through sexual reproduction across the entire population [16,17,18]. In nature, these selfish genes manipulate on order to favor their transmission even at the expense of fitness costs [17,19,20]. Although this unique property has garnered the attention of weed scientists, the new surge in interest is attributed to the adoption of the revolutionary technology, i.e., ‘CRISPR-Cas9’ (Clustered Regularly Interspaced Short Palindromic Repeats—CRISPR-associated nucleases) in gene drive systems [15,17,18,21,22]. CRISPR-Cas9 technology empowers gene editing with unparalleled accuracy and specificity by leveraging the guide RNA (gRNA) sequence and Cas9 endonuclease complex to precisely locate and cleave the target site [23,24,25]. Considering the potential benefits, this article explores the role of gene drive and highlights the versatility of CRISPR-Cas9 in effective weed management.

## 2. Gene Drive Systems

**Figure 1:**
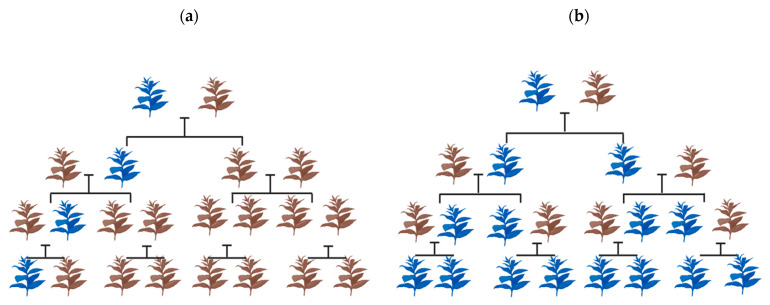
Spread of the allele containing the drive in a population. (**a**) Normal inheritance pattern in the absence of a drive allele. (**b**) Gene drive-mediated inheritance, where the altered gene bias influences the inheritance pattern. The brown plant represents the wild type of individual while the blue represents the individual with the gene drive allele.

In natural wild populations, Mendelian genetics offers equal opportunity for both the contributing alleles of a gene to be transmitted to the offspring during sexual reproduction (Figure 1a). With gene drive, an allele harboring the drive preferably has a superior chance of being inherited compared to the wild type. In due course, the drive proliferates gradually over segregating generations, with a theoretical possibility of achieving a homogeneous population over multiple generations (Figure 1b). This property has rendered gene drives with enhanced transmission, and thus, they are often labeled as ‘selfish genetic elements’ [19]. Their impact, however, may be neutral or detrimental to the organism [26,27,28]. Naturally, gene drives thrive via mobile transposable elements, maternal effect dominant embryonic arrest (Medea), meiotic drive, and homing endonuclease genes (HEGs) [23,29]. The mechanism by which these genetic elements drive is essentially different from the other.

### 2.1. Transposable Elements

Transposable elements—also known as ‘jumping genes’—are one of the earliest known selfish genes that relocate themselves to different parts of the genome. They contribute to almost 50–80% of some plant genomes [30]. Although initially considered to be junk DNA with little function, they were later recognized to control the population of *Drosophila melanogaster* [31]. The ability of transposable elements to drive has been demonstrated by introducing autonomous and non-autonomous P element constructs over 40 generations in *D. melanogaster* [32]. While the feasibility of using transposable elements as a driver was shown in *Anopheles* species for the control of malaria and dengue in simulated models, they failed to be an efficient driver due to the larger fitness cost generated by non-specific re-mobilization into new positions of the genome [33,34].

### 2.2. Medea

Medea are autosomal factors that make it obligatory for them to be transmitted to the offspring by eliminating those that do not inherit the Medea allele [35,36]. Thus, offspring with Medea will survive, while those without it will die due to maternal lethality coupled with zygotic self-rescue, a ‘maternal-toxin/zygotic-antidote system’. The toxin and antidote are tightly linked in Medea-bearing mothers. Mating between a normal female and heterozygous Medea male produces wild-type and Medea-bearing progenies. Conversely, mating between heterozygous Medea females and wild-type males only allows Medea-inherited progenies to survive [36,37,38,39,40]. The gene drive potential of Medea was documented in *D. melanogaster* by targeting maternally expressed genes such as *myd88* [37,39], *dah*, and *o-fut1* [41]. This was achieved by encoding a microRNA (miRNA), which functions as the toxin, and a recoded sequence of the target that is insensitive to the miRNA as the antidote under suitable promoters [37,39,41]. Medea prototypes as gene drives have been tested in caged experiments and under field conditions. However, developing an effective miRNA toxin/antidote system is challenging and is a major hurdle to exploring Medea’s application in organisms other than *D. melanogaster*. The dynamic nature of Medea’s distribution in flour beetle (*Tribolium castaneum*) in the United States has been analyzed by tracing two Medea elements, M1 and M4, across beetle populations. This has given insight into their co-existence and preliminary evidence of the non-uniform spread of Medea in target species with limited dispersal [40].

### 2.3. Meiotic Drive

Meiotic drive is a combination of various genetic elements such as paternal genome eliminators, spore killers, supernumerary B chromosomes, female meiotic drive, and male meiotic drive [42,43,44,45,46,47,48]. These elements are grouped into two categories: true or pre-gametic and killer or post-gametic meiotic drives, based on mechanistic and temporal differences in their modes of action [49].

True or pre-gametic drive occurs during meiosis to favor transmission of the drive-bearing allele in the gamete [50,51]. The female meiotic drive is the most studied pre-gametic drive. Meiosis in female flowering plants prefers only one of the four meiotic products to become a mature gametophyte while the remaining three form polar bodies that eventually degenerate [52]. This asymmetry creates a genetic conflict among the homologous chromosomes as they compete to form the egg cell [16,43], as observed in maize (*Zea mays* L.) [53,54,55] and yellow monkey flower (*Mimulus guttatus*) [56]. In maize, the ‘knob’ 51 is formed by repeating sequences on the abnormal chromosome 10, ‘Ab10’. Ab10 uses these knobs to move towards the spindle pole by creating neocentromeres instead of regular centromeres, which results in preferential segregation. This mobility was later attributed to the kinesin driver (Kindr) and Tr-1 Kinesin (TRKIN) complexes [53,55].

The killer meiotic drive occurs post meiosis and is tagged as ‘ultra-selfish’ due to its destructive nature. The killer drive sabotages meiotic products that do not inherit the drive in both males and females [50]. Killer meiotic drives can be either indiscriminate or selective. Indiscriminate killers use a “killer-target” system, while selective killers use a “toxin-antidote” mechanism [49]. One example is the segregation distorter (SD) in *D. melanogaster*, which results in the survival of only the spermatids inheriting the drive allele [57,58,59,60]. Another example is sex chromosome-based meiotic drive, which promotes a male or female dominant progeny population, leading to skewed sex ratios [61]. A Y-drive system in *Anopheles gambiae* destroyed X-chromosomes during spermatogenesis and produced 95% male progeny [62]. The selective killers that work on the toxin–antidote system were identified in rice loci qHMS7 and OsCOX11, where genes *ORF2* and *ORF3* for locus qHMS7, and *DUYAO* and *JIEYAO* for locus OsCOX11, encoded for toxins and antidotes, respectively [63,64].

### 2.4. Homing Endonuclease Genes (HEGs)

HEGs exploit the cell’s repair machinery by creating a double-strand break in the homologous chromosome devoid of HEG, through its site-specific endonuclease gene. The HEG then acts as a savior by introducing the HEG-containing sequence as a template for repair via a homology-directed repair mechanism. Thus, the HEG-template duplicates by substituting induced breaks in the genome in a process called ‘homing’ to favor their opportunistic selfish transmission [19,65,66]. HEGs are distributed across the intervening sequences at the RNA (intron) and protein (intein) levels and employ a self-splicing mechanism to avoid unsolicited interference with the host genome [67]. Their potential as a drive was confirmed by cage experiments in *A. gambiae* for the control of malaria and dengue [68]. The HEG proteins, which have a nominal size of less than 40 kDa, are specific and mobile, making them ideal for targeted genome editing [67,69,70]. Undoubtedly, they are a rapidly emerging area of innovation. Of all of the naturally occurring functional gene drive systems discussed so far, HEGs have been the most extensively reviewed due to a broader understanding of the molecular mechanisms and ease in manipulating the drive system to suit the host [71,72]. Their success has inspired the likelihood of employing other genome editing technologies including ZFN (zinc finger nucleases), TALEN (transcription activator-like effector nucleases) and CRISPR-Cas9 in gene drive systems.

## 3. CRISPR: A Biotechnological Breakthrough in Artificial Gene Drive

CRISPR has revolutionized targeted genome editing ever since its conception [73,74,75,76]. It was originally discovered in bacteria as an adaptive immune system and protects against invading phages through RNA-guided DNA cleavage [73]. This is achieved in a three-step process: adaptation (the unique sequence specific to the invading foreign particle called a ‘protospacer’ is integrated into the CRISPR locus of the host bacterial genome), biogenesis (the sequence undergoes transcription to produce a mature guide RNA sequence), and interference (matching between the mature guide RNA and invading target sequence activates cleavage of the target by the endonuclease enzyme) [77,78]. Thus, two fundamental components, the gRNA sequence (a short nucleotide sequence of preferably 17–20 bp) and the Cas9 endonuclease characterize genome editing by CRISPR [79], resulting in a mosaic of mutations including deletions, insertions, and substitutions [75,77]. The activity of Cas9 is dependent on a short sequence of 3–6 base pairs called a protospacer adjacent motif (PAM), which is located downstream of the target sequence [77]. CRISPR/Cas9’s application in gene drive is favored by their high specificity and flexibility for targeting any host organism irrespective of species [23]. Its principle is similar to that of HEGs with an additional benefit of ease in designing a synthetic drive [23,80].

**Figure 2:**
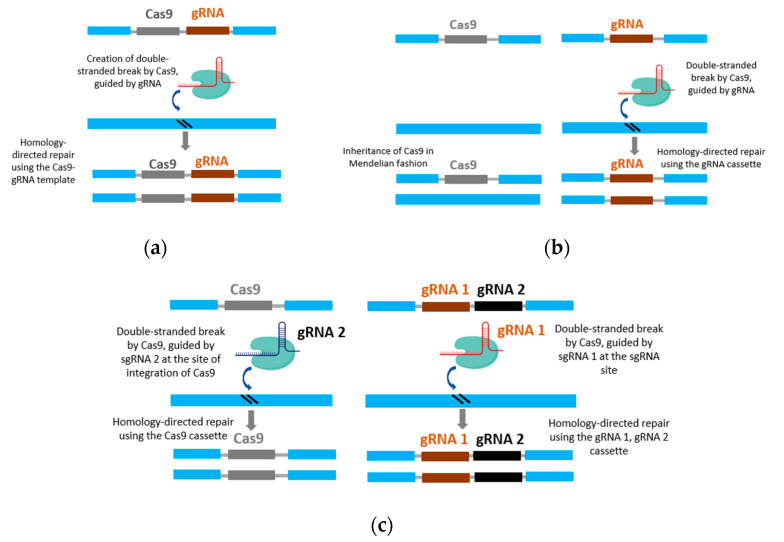
CRISPR-based gene drive system. (**a**) Full gene drive system with Cas9 and gRNA elements in a single cassette. The gRNA directs the Cas9 to cleave the target site in the genome. The fGD cassette is used as a template for homology-directed repair. (**b**) Simple split gene drive system with Cas9 and gRNA elements in two different gene cassettes. The cassette harboring the Cas9 is transmitted in Mendelian fashion, while the cassette with the gRNA is copied in the genome. (**c**) Trans-complimenting gene drive with two gRNAs, with an additional gRNA2 to direct cleavage at the Cas9 insertion site. This allows both of the cassettes to be copied in the genome.

The CRISPR gene drive element consists of two important components: Cas9, which provides the endonuclease activity necessary to function as an HEG, and the gRNA, which directs Cas9 to make the site-specific double-stranded break on the target genome. During the repair process of the double-stranded break, the chromosome bearing the Cas9 and gRNA cassette is used as a template for homology-directed repair (HDR), thereby enabling the introduction of the desired trait at a specific locus of the genome [23,81]. In addition to the Cas9 and gRNA, any gene placed next to the Cas9 can also be replicated during the HDR process. This allows the CRISPR-Cas9 gene drive to carry a “cargo” unit or transgene that contains a desired trait of interest [81]. Depending on the arrangement of Cas9 and gRNA, either in a single cassette or in different cassettes, CRISPR gene drives can be classified into two groups: full gene drives (fGDs) and split gene drives (sGDs). In fGD, the gRNA directs the Cas9 present in the same cassette to cleave at the target site and is often called a ‘linked gene drive’ (Figure 2a). In the sGD method, the cassettes containing the gRNA and Cas9 are inserted at different locations in the genome (Figure 2b). The gRNA is placed at the site where Cas9 cleaves the DNA, while Cas9 is inserted elsewhere in the genome. The gRNA cassette is functional only when it is present along with the Cas9 cassette. The Cas9 cassette is inherited in Mendelian fashion, while the gRNA cassette is copied in an additive manner. However, in the absence of the Cas9 cassette, the gRNA cassette is also inherited in Mendelian fashion [82]. The sGD system allows to evaluate the individual effects of each component, the Cas9 or the gRNA in directing the drive, resulting in better control options [83]. There is a more sophisticated type of sGD, known as trans-complementing drive, which includes a second gRNA that cleaves the insertion site of the Cas9 (Figure 2c). The inclusion of this second gRNA enables both the Cas9 and gRNA cassettes to function as full gene drives in trans during a genetic cross, such that both of the components are inherited as gene drive elements [84].

Simulated models for the use of CRISPR/Cas9 as an effective synthetic gene drive to control pests have been attempted, with a primary focus on controlling malaria [22,24,85]. Synthetic gene drives were designed with gRNA constructs targeting the recessive gene responsible for female sterility in the malarial vector, *A. gambiae*. Knock-out female mutants that were generated failed to produce offspring, resulting in a predominantly sterile female population within four generations. This substantially reduced the transmission of the disease [24]. A similar approach was used to target, *AgdsxF*, a gene that affects female development in *A. gambiae* [22]. These caged experiments have proven to be pragmatically reliable techniques to solve the menace of malaria. CRISPR/Cas9’s competence as a drive was also examined to control *Aedes aegypti*, the vector responsible for Zika virus, yellow fever, dengue, and chikungunya, to design a synthetic drive that is safe for release into a wild population [86].

Although CRISPR/Cas9 follows the principle of HEG, it can be successfully employed to perform like other gene drive systems such as sex chromosome meiotic drive [87] and Medea [42]. This is because all gene drive systems require some form of impairment that interferes with the development of the competing allele. For instance, a Y-drive system using CRISPR was used to target a conserved ribosomal DNA sequence located on the X-chromosome, thereby favoring a male biased progeny with 86.1% to 94.8% males to control malaria [87]. Also, Medea-based CRISPR termed as ‘TARE’ (toxin–antidote recessive embryo) was successful in modifying a relatively small regional population of *D. melanogaster* by targeting a recessive lethal gene. TARE-CRISPR Cas9 provides an additional advantage of slowing down the formation of resistant alleles. The TARE drive was designed with a portion of the gene that can restore its function in the event of resistance [88]. These successful lab experiments demonstrate the ability of CRISPR technology and have generated enthusiasm for its potential use in wild populations.

## 4. Factors Governing the Success of Gene Drive for Weed Management

Considering the lack of empirical data to demonstrate the application of gene drive in plants, success stories in animal systems have been studied to understand their mechanism of action. In insects, the choice of drive system determines whether one can aim to eliminate the pest population or instead modify it to render them more susceptible to toxins [17,23,89]. Similarly, to manage weeds, synthetic gene drives can be utilized using two approaches: population suppression and population modification or sensitization [15]. In population suppression, drives that engender lethality in weed species like killer meiotic drive can be adopted [22,49]. These drives can be used to target major genes in weeds responsible for the growth, development, and survival of the weed species. For population modification or sensitization, one can use drives that disrupt the weed genome, rendering them more vulnerable to weed management practices [16], for example, HEG drive systems [17]. A proposal for population modification to make weeds more susceptible to herbicide application has been suggested [16]. Another potential is reversing herbicide-resistant weeds to their natural forms so that herbicides can effectively target them again [15]. This will minimize the constant need to develop herbicides that target novel sites of action [90]. To translate these concepts into reality, thorough knowledge of the reproductive biology of weeds, fitness, seed banks, and response to herbicidal applications besides acknowledging the molecular basis of synthetic drive is needed [18]. These factors will determine the acceptance and persistence of gene drives in weed ecosystems.

### 4.1. Mode of Reproduction in Weed Species

Weed biology is a diverse field that covers a wide range of topics such as weed morphology, growth, reproduction, and their life cycle [91]. The mode of reproduction plays a critical role, as gene drives are more effective in individuals that reproduce sexually [92]. The majority of weed species have an asexual mode of reproduction coupled with a perennial life cycle which make gene drive’s application a challenge [15]. However, some of the notorious annual weed species like kochia (*Bassia scoparia*), Palmer amaranth (*Amaranthus palmeri*), waterhemp (*Amaranthus tuberculatus*)*,* and others reproduce through seeds, which make them ideal candidates for gene drive [93,94,95]. A thorough understanding of the reproductive biology and mating patterns of the target species is crucial in accurately predicting the critical parameters required for the successful release of gene drive technology. This includes determining the precise number of gene drive organisms (GDOs) needed for a successful outcome [96].

To more effectively target weeds such as *A. palmeri* and *A. tuberculatus* [97], which are dioecious in nature, sex chromosome-based drive systems could prove useful. Utilizing gene drive technology may be most efficient when focusing on weeds with reproductive traits that favor sexual hermaphroditism, like protandry and protogyny. In cases where meiotic drive systems are a viable option, this could be a successful approach with a deep understanding of the molecular mechanisms of these traits. The identification of male specific sequence in *A. tuberculatus* and *A. palmeri* will help in developing a male-dominated population so that the seed bank reserve is greatly reduced [97,98]. Researchers have also explored the possibility of utilizing gene drives to constrain the phenotypic expression of certain traits. For example, homologs of wheat Rht dwarfing alleles can be screened in tall weed species like ragweed, which compete with crop species for light [15,99].

The life cycle of weeds decides how fast a gene drive can fix into a population. An annual weed like *A. palmeri*, is estimated to take 15–20 generations to get a drive allele successfully fixed into a population based on mathematical models, as suggested earlier [89]. This also depends on the seed bank, as they are the primary source of infestation of annual weeds and vary between different cropping systems [100]. The viability of weed seeds in a corn field indicated 10–50% [91]. These seeds may emerge under favorable conditions and might interfere with the proposed model of progression of a drive allele.

### 4.2. Establishment of the International Weed Genomics Consortium and the Genomic Database of Weeds

To create suppression and modification gene drives, genes that are responsible for fitness, competition, and adaptation to different agroecosystems can be targeted. However, this requires genomic information of the target genes. The International Weed Genomics Consortium (IWGC) has developed a database of weed species called WeeDpedia (https://www.weedgenomics.org/weedpedia/ (accessed on 20 October 2023)). Currently, WeeDpedia has genome assemblies for 31 weed species, including those causing significant economic losses like *A. palmeri*, *A. tuberculatus*, and *Avena fatua*. Although the sequences of some weed species are not yet available in the public domain, they are in progress. The database has helped in identifying sex-linked markers in *A. tuberculatus* which are desirable to serve as drives [97]. Thus, the success of gene drive technology depends on the amount of genomic information available for each weed species. Fortunately, with advancements in sequencing technology, it has become easier and more cost-effective to sequence non-model organisms. Therefore, weed science research can contribute to the growth of the IWGC database by leveraging this opportunity.

The reversal of herbicide resistant traits in weeds into wild forms with gene drive requires extensive information on their evolutionary genetics. Resistance arises either due to direct modification of the target allele (target site resistance, TSR) or indirect alterations in the non-target alleles that collectively impair herbicidal action (non-target site resistance, NTSR) [101]. A plethora of articles devoted towards understanding these mechanisms are available. TSR and NTSR can impose resistance development, either independently or mutually. The majority of TSR results from a single nucleotide polymorphism (SNP) in the target gene [102]. For example, SNPs causing amino acid changes of Ser164Gly and Ser264Thr are responsible for resistance to triazine group of herbicides [103,104]. These specific target positions can be edited using a CRISPR-based drive system to revert them to their wild form.

## 5. CRISPR-Cas9 Gene Drive for Weed Control

Access to the target organism’s genomic sequence is crucial when creating a guide RNA (gRNA) for CRISPR-Cas9 gene drive. There are web-based gRNA design tools, such as CRISPR Plant (http://omap.org/crispr/ (accessed on 23 October 2023)) and CRISPR P 2.0 (http://crispr.hzau.edu.cn/CRISPR2/ (accessed on 23 October 2023)), available for various plant species. These tools provide a wide range of PAM sites to choose from, prioritize specificity (on-target score), and minimize off-target scores [105]. They can even be adapted for weeds. CRISPR is the most realistic drive system available, and its ability to enhance the spread of gene drive has been extensively studied in models like mutagenic chain reaction (MCR) and daisy chain reaction [83,106,107]. The MCR model allows gene drive to spread rapidly through the population by producing homozygous mutants through autocatalytic mutations. However, uncontrolled spread without any limiting factor could result in resistant alleles that would eventually overcome the gene drive. The daisy chain reaction model, on the other hand, uses split gene drives to slow down the rate of resistant allele formation. It consists of a linear arrangement of unlinked genetic elements, with those at the base of the chain losing the drive function and being eliminated via natural selection [107]. This prevents a sudden increase in drive-bearing individuals that could shock the population.

The ground-breaking discovery of a sex-linked genomic sequence in the Y-chromosomal regions of *A. palmeri* and *A. tuberculatus* that determines dioecy is a significant contribution [97,98]. A sex-linked meiotic drive system using CRSIPR/Cas9 can be introduced to produce a skewed male-dominated population [24]. For the release of such individuals engineered with drive into a wild population, the major risk of evolved resistance should always be considered. Various mathematical models for designing the nature of release, aimed at reducing resistance, have been proposed, with targets to modify a small local population first and then allowing them to spread gradually. An example is that of a threshold-dependent drive system, in which the drive thrives only when released above a threshold value [108].

The TSR mechanism of herbicide resistance in weeds, which has been largely attributed to SNPs [102] and transmitted as single dominant mutations [109,110], provides scope for the use of CRISPR-based gene drive. CRISPR gene drive can be employed to revert the resistant population into its wild form by designing gRNAs encompassing the SNP position. Resistance involving NTSR will require the multiplexing of gRNAs to confer the desired trait. This proposal of reversing the resistant gene’s function into normalcy eliminates the factor of resistance development, which is a limiting feature associated with gene drive. Under such circumstances, an MCR-based drive system is preferable, since the allele bearing the drive will indiscriminately spread into a population within a short period [89]. Thus, the population harboring the wild-type gene can be obtained, whereby the same herbicide will be as effective as before. This will save us from the relentless search for novel target sites to develop herbicides.

Although CRISPR has enormous potential, its practicality is often constrained by the lack of an effective transformation system for the target weed species. To apply CRISPR-Cas9 technology to weed species, a well-established tissue culture protocol for each species needs to be developed, followed by a standardized transformation protocol. However, there are limited reports of any tissue culture protocols for weed species. Some weed species have complex or poorly understood physiological characteristics, which makes tissue culture more challenging. They may have unique requirements for growth factors, hormones, or environmental conditions that are not easily replicable in vitro. Certain weed species may be recalcitrant to tissue culture, meaning that they are resistant to in vitro propagation. This resistance can be due to factors such as the production of secondary metabolites, a lack of responsive tissues, or genetic barriers. Additionally, weed species exhibit high genetic diversity, and tissue culture techniques may not work uniformly across all genotypes within a species, making it challenging to develop broadly applicable tissue culture methods. The lack of tissue culture and transformation protocols may therefore hinder the progress of applying CRISPR-Cas9 technology in gene drive. Recently, our group optimized a regeneration protocol via callus culture in waterhemp (Kumam et al. minor revision in review). Work is in progress to establish similar protocols in other weeds such as Palmer amaranth and kochia.

## 6. CRISPR-Modified Crop Plants for Herbicide Tolerance

Weed management is not only restricted to the use of effective herbicides but also used in the development of herbicide-resistant crops. Genetic engineering’s contribution has been especially tremendous in imparting this trait to major crops like corn/maize, soybean (*Glycine max* L. Merril) and cotton (*Gossypium* sp.). A total of 349 herbicide-resistant transgenic crops have been made publicly available as of now with corn contributing the highest percentage of 86.50. The majority of these crops are engineered for glyphosate resistance by targeting the *EPSPS* gene (http://www.isaaa.org/gmapprovaldatabase/ (accessed on 24 October 2023)). With genome editing tools at our disposal, the subject has gained more momentum. Genome-editing to impart herbicide resistance in crops has been attempted in maize, watermelon (*Citrullus lantus* L.), and oilseed rape (*Brassica napus* L.) to impart resistance against ALS (acetolactate synthase), inhibiting herbicides by base editing the *ALS* gene; in soybean to impart resistance to AHAS (acetohydroxyacid synthase), inhibiting herbicides by editing *AHAS* gene; in rice to impart resistance to glyphosate, th eALS inhibitor, the ACCase (Acetyl CoA carboxylase) inhibitor, and triketone herbicides by targeting *EPSPS*, *ALS*, *ACCase* and *HPPD* genes, respectively [111,112,113,114,115,116,117].

Whilst the notion of producing crops that can withstand herbicides has been widely embraced, when viewed pragmatically, it does not necessarily reduce the dose and cost of herbicide application in the field [90,118]. As such, a dual approach involving the use of herbicide-tolerant crops along with gene drives that modify them for weed populations would prove to be a highly effective weed management strategy.

## 7. Risk Aspects

Unprecedented risks associated with the administration of gene drive in wild populations are a key factor limiting its application. The evolution of resistance against the drive is one such area of concern. In CRISPR drive systems, the Cas9-induced cleavage of the target region is predominantly repaired by non-homologous end joining (NHEJ) pathways rather than HDR [24,85]. This results in a modified target site that is often unrecognizable to the drive for inducing subsequent cleavage reactions [119]. Often, this has led to the identification of resistant alleles in lab experiments using HEGs within a short period of 1–2 generations, which is quite alarming as they can easily spread over longer time periods [24,119,120]. A way to overcome this is the use of trans complementing gene drive, where the gRNA and Cas9 elements are inherited individually in Mendelian fashion, while they function as a full drive only in combination [121]. Gene drive systems that rely on homing and meiosis can become widespread in a population even with the release of only a small number of individuals carrying the gene drive, as long as the fitness costs of carrying the gene drive do not outweigh the inheritance advantage provided by the gene drive itself [108]. A method to reduce this rate of resistant allele formation is the multiplexing of gRNAs to delay the evolution of resistance [106]. HEG-based drives also possess the ability of horizontal gene transfer [19]. This will permit the spread of drive to non-target crop species of economic importance. Several factors contribute to the potential transfer of gene drive and the contamination of non-target species. DRAQUE’s (Drive Risk Assessment Quantitative Estimate) equation has been used to calculate these factors, which includes hybridization between target and non-target species, Cas9 and gRNA expression, recognition of the target sequence cleaved in non-target species, the insertion of the gene drive cassette at the cleavage site, the acceptance of Cas9 expression by the host’s immune cells, and the invasive nature of the drive within the population [122]. Palmer amaranth, a weed that is highly similar to its cultivated species such as *A. caudatus*, *A. cruentus*, and *A. hypochondriacus*, is likely to escape gene drive-bearing alleles through outcrossing, which could disrupt the ecological balance and cause irreversible damage to the cultivated species. Similarly, invasive weeds like ragweed have a high outcrossing ability and thus require extensive simulation models to identify their spread before release into wild populations. Thus, it is always necessary to perform experiments with weed species under contained greenhouse or growth chamber conditions, with a preset number of individuals carrying the drive-bearing allele and wild population, to see the degree of spread of the drive element, while simultaneously monitoring the nature of resistance evolution, not only in the target weed species, but also in non-target species which are closely related. Additionally, a major limiting factor for gene drives is the fitness cost associated with them. A gene drive’s persistence in wild populations is largely determined by the fitness cost associated with it. Even if a gene drive is efficient, it may have a chance of being eliminated in the wild due to the large fitness cost involved during natural selection [123].

## 8. Conclusions

Although the genetic control of weeds is vital, considering the urgent need for sustainable solutions, the technology remains a subject of controversy in public, in academia, and among policy-makers [124]. While designing a gene drive, it is imperative to consider the ecological, social, political, and ethical concerns concomitant with the employment of the gene drive system under field conditions [20,125]. The application of gene drive has the potential to significantly impact the natural ecosystem of a local population [126]. Therefore, it is crucial to exercise extreme caution when conducting experiments and be prepared for any unforeseen consequences that may arise. Appropriate measures should be taken to prevent any negative effects on the ecosystem, using proper risk assessment and management approaches. It is imperative to treat gene drive-related techniques on a case-by-case basis. This will ensure that the guidelines and regulatory frameworks outlined serve the specific needs of the local area where the gene drive is set to be released [127]. This requires the involvement of scientific experts, local experts, field site practitioners, and community leaders in debates and discussions in topics related to gene drive research and possible release into the wild [127]. It is crucial that all stakeholders work together to evaluate the pros and cons of using gene drive technology to modify or suppress pest populations, given the uncertainty surrounding its practical application. Transparency in gene drive research is of utmost importance, and findings should be made readily accessible to the public to ensure that local communities are aware of the potential consequences associated with the release of organisms carrying gene drives [128]. With respect to the management of insect pests, the “Gene Convene Global Collaborative” (https://fnih.org/our-programs/geneconvene-global-collaborative/ (accessed on 20 November 2023)) has taken the initiative to address the issues regarding deployment and research concerning gene drive. Exemption of CRISPR-generated mutants from being regulated under transgenic crops in a few countries including the USA, Israel, Japan, New Zealand, and Australia favors the implementation of a CRISPR-Cas9-based gene drive system [129]. Nevertheless, the possibility of gene drives that perform well in simulated models being outperformed by their wild-type counterparts under field conditions cannot be entirely ruled out. How efficient and fast gene drive can spread into weed populations to cause population suppression or modification needs to be supplemented with empirical data from major notorious weeds. To achieve effective weed management using gene drives, it is crucial to receive support from various fields such as genomics, weed biology, drive mechanisms, and regulatory authorities. While it may seem a long shot, collaborative efforts between these fields will pave the way for gene drives to become one of the most competent technologies in weed management.

## Data Availability

Not applicable.

## References

[1] Oerke E.-C. (2006). Crop Losses to Pests. J. Agric. Sci..

[2] Dayan F.E. (2019). Current Status and Future Prospects in Herbicide Discovery. Plants.

[3] (2023). Heap Current Status of the International Herbicide-Resistant Weed Database. www.weedscience.org.

[4] Bajwa A.A. (2014). Sustainable Weed Management in Conservation Agriculture. Crop Prot..

[5] Cordeau S., Triolet M., Wayman S., Steinberg C., Guillemin J.P. (2016). Bioherbicides: Dead in the Water? A Review of the Existing Products for Integrated Weed Management. Crop Prot..

[6] Singh H.P., Batish D.R., Kohli R.K. (2003). Allelopathic Interactions and Allelochemicals: New Possibilities for Sustainable Weed Management. CRC Crit. Rev. Plant Sci..

[7] Tesio F., Ferrero A. (2010). Allelopathy, a Chance for Sustainable Weed Management. Int. J. Sustain. Dev. World Ecol..

[8] Baltzegar J., Cavin Barnes J., Elsensohn J.E., Gutzmann N., Jones M.S., King S., Sudweeks J. (2018). Anticipating Complexity in the Deployment of Gene Drive Insects in Agriculture. J. Responsible Innov..

[9] Khanh T.D., Xuan T.D., Chung I.M. (2007). Rice Allelopathy and the Possibility for Weed Management. Ann. Appl. Biol..

[10] Auld B.A., Hetherington S.D., Smith H.E. (2003). Advances in Bioherbicide Formulation. Weed Biol. Manag..

[11] Kremer R.J. (2005). The Role of Bioherbicides in Weed Management. Biopestic. Int..

[12] Bhowmik P.C. (2003). Inderjit Challenges and Opportunities in Implementing Allelopathy for Natural Weed Management. Crop Prot..

[13] Curtis C.F. (1985). Genetic Control of Insect Pests: Growth Industry or Lead Balloon?. Biol. J. Linn. Soc..

[14] Gould F. (2008). Broadening the Application of Evolutionarily Based Genetic Pest Management. Evolution.

[15] Neve P. (2018). Gene Drive Systems: Do They Have a Place in Agricultural Weed Management?. Pest Manag. Sci..

[16] Barrett L.G., Legros M., Kumaran N., Glassop D., Raghu S., Gardiner D.M. (2019). Gene Drives in Plants: Opportunities and Challenges for Weed Control and Engineered Resilience. Proc. R. Soc. B Biol. Sci..

[17] Champer J., Buchman A., Akbari O.S. (2016). Cheating Evolution: Engineering Gene Drives to Manipulate the Fate of Wild Populations. Nat. Rev. Genet..

[18] Kumaran N., Choudhary A., Legros M., Sheppard A.W., Barrett L.G., Gardiner D.M., Raghu S. (2020). Gene Technologies in Weed Management: A Technical Feasibility Analysis. Curr. Opin. Insect Sci..

[19] Burt A. (2003). Site-Specific Selfish Genes as Tools for the Control and Genetic Engineering of Natural Populations. Proc. R. Soc. B Biol. Sci..

[20] Wedell N., Price T.A.R., Lindholm A.K. (2019). Gene Drive: Progress and Prospects. Proc. R. Soc. B Biol. Sci..

[21] Bull J.J., Malik H.S. (2017). The Gene Drive Bubble: New Realities. PLoS Genet..

[22] Kyrou K., Hammond A.M., Galizi R., Kranjc N., Burt A., Beaghton A.K., Nolan T., Crisanti A. (2018). A CRISPR–Cas9 Gene Drive Targeting Doublesex Causes Complete Population Suppression in Caged Anopheles Gambiae Mosquitoes. Nat. Biotechnol..

[23] Esvelt K.M., Smidler A.L., Catteruccia F., Church G.M. (2014). Concerning RNA-Guided Gene Drives for the Alteration of Wild Populations. eLife.

[24] Hammond A., Galizi R., Kyrou K., Simoni A., Siniscalchi C., Katsanos D., Gribble M., Baker D., Marois E., Russell S. (2016). A CRISPR-Cas9 Gene Drive System Targeting Female Reproduction in the Malaria Mosquito Vector Anopheles Gambiae. Nat. Biotechnol..

[25] DiCarlo J.E., Chavez A., Dietz S.L., Esvelt K.M., Church G.M. (2015). Safeguarding CRISPR-Cas9 Gene Drives in Yeast. Nat. Biotechnol..

[26] Hurst G.D.D., Werren J.H. (2001). The Role of Selfish Genetic Elements in Eukaryotic Evolution. Nat. Rev. Genet..

[27] Werren J.H., Nur U., Wu C.I. (1988). Selfish Genetic Elements. Trends Ecol. Evol..

[28] Werren J.H. (2011). Selfish Genetic Elements, Genetic Conflict, and Evolutionary Innovation. Proc. Natl. Acad. Sci. USA.

[29] Burt A., Crisanti A. (2018). Gene Drive: Evolved and Synthetic. ACS Chem. Biol..

[30] Feschotte C., Jiang N., Wessler S.R. (2002). Plant Transposable Elements: Where Genetics Meets Genomics. Nat. Rev. Genet..

[31] Ribeiro J.M., Kidwell M.G. (1994). Transposable Elements as Population Drive Mechanisms: Specification of Critical Parameter Values. J. Med. Entomol..

[32] Carareto C.M.A., Kim W., Wojciechowski M.F., O’Grady P., Prokchorova A.V., Silva J.C., Kidwell M.G. (1997). Testing Transposable Elements as Genetic Drive Mechanisms Using Drosophila P Element Constructs as a Model System. Genetica.

[33] Rasgon J.L., Gould F. (2005). Transposable Element Insertion Location Bias and the Dynamics of Gene Drive in Mosquito Populations. Insect Mol. Biol..

[34] Marshall J.M. (2008). The Impact of Dissociation on Transposon-Mediated Disease Control Strategies. Genetics.

[35] Beeman R.W., Friesen K.S., Denell R.E. (1992). Maternal-Effect Selfish Genes in Flour Beetles. Science.

[36] Beeman R.W., Friesen K.S. (1999). Properties and Natural Occurrence of Maternal-Effect Selfish Genes (“Medea” Factors) in the Red Flour Beetle, Tribolium Castaneum. Heredity.

[37] Chen C.-H., Huang H., Ward C.M., Su J.T., Schaeffer L.V., Guo M., Hay B.A. (2007). A Synthetic Maternal-Effect Selfish Genetic Element Drives Population Replacement in Drosophila. Science.

[38] Ward C.M., Su J.T., Huang Y., Lloyd A.L., Gould F., Hay B.A. (2011). Medea Selfish Genetic Elements as Tools for Altering Traits of Wild Populations: A Theoretical Analysis. Evolution.

[39] Buchman A., Marshall J.M., Ostrovski D., Yang T., Akbari O.S. (2018). Synthetically Engineered Medea Gene Drive System in the Worldwide Crop Pest Drosophila Suzukii. Proc. Natl. Acad. Sci. USA.

[40] Cash S.A., Lorenzen M.D., Gould F. (2019). The Distribution and Spread of Naturally Occurring Medea Selfish Genetic Elements in the United States. Ecol. Evol..

[41] Akbari O.S., Chen C.-H., Marshall J.M., Huang H., Antoshechkin I., Hay B.A. (2014). Novel Synthetic Medea Selfish Genetic Elements Drive Population Replacement in Drosophila; a Theoretical Exploration of Medea-Dependent Population Suppression. ACS Synth. Biol..

[42] Lindholm A.K., Dyer K.A., Firman R.C., Fishman L., Forstmeier W., Holman L., Johannesson H., Knief U., Kokko H., Larracuente A.M. (2016). The Ecology and Evolutionary Dynamics of Meiotic Drive. Trends Ecol. Evol..

[43] Jones R.N. (1995). B Chromosomes in Plants. New Phytol..

[44] Houben A., Banaei-Moghaddam A.M., Klemme S., Timmis J.N. (2014). Evolution and Biology of Supernumerary B Chromosomes. Cell. Mol. Life Sci..

[45] Yoshida K., Kitano J. (2012). The Contribution of Female Meiotic Drive to the Evolution of Neo-Sex Chromosomes. Evolution.

[46] Presgraves D., Birkhead T.R., Hosken D.J., Pitnick S. (2009). Drive and Sperm: The evolution and genetics of male meiotic drive. Sperm Biology: An Evolutionary Perspective.

[47] Raju N.B. (1994). Ascomycete Spore Killers: Chromosomal Elements that Distort Genetic Ratios among the Products of Meiosis. Mycologia.

[48] Úbeda F., Normark B.B. (2006). Male Killers and the Origins of Paternal Genome Elimination. Theor. Popul. Biol..

[49] Angélica M., Núñez B., Nuckolls N.L., Zanders S.E. (2019). Genetic Villains: Killer Meiotic Drivers. Trends Genet..

[50] Zimmering S., Sandler L., Nicoletti B. (1970). Mechanisms of Meiotic Drive. Annu. Rev. Genet..

[51] Courret C., Chang C.H., Wei K.H.C., Montchamp-Moreau C., Larracuente A.M. (2019). Meiotic Drive Mechanisms: Lessons from Drosophila. Proc. R. Soc. B Biol. Sci..

[52] Yang W.-C., Shi D.-Q., Chen Y.-H. (2010). Female Gametophyte Development in Flowering Plants. Annu. Rev. Plant Biol..

[53] Dawe R.K., Lowry E.G., Gent J.I., Stitzer M.C., Swentowsky K.W., Higgins D.M., Ross-Ibarra J., Wallace J.G., Kanizay L.B., Alabady M. (2018). A Kinesin-14 Motor Activates Neocentromeres to Promote Meiotic Drive in Maize. Cell.

[54] Buckler E.S., Phelps-Durr T.L., Buckler C.S.K., Dawe R.K., Doebley J.F., Holtsford T.P. (1999). Meiotic Drive of Chromosomal Knobs Reshaped the Maize Genome. Genetics.

[55] Swentowsky K.W., Gent J.I., Lowry E.G., Schubert V., Ran X., Tseng K.-F., Harkess A.E., Qiu W., Dawe R.K. (2020). Distinct Kinesin Motors Drive Two Types of Maize Neocentromeres. Genes Dev..

[56] Fishman L., Willis J.H. (2006). A Cytonuclear Incompatibility Causes Anther Sterility in Mimulus Hybrids. Evolution.

[57] Sandler L., Hiraizumi Y., Sandler I. (1959). Meiotic Drive in Natural Populations of Drosophila Melanogaster. I. The Cytogenetic Basis of Segregation-Distortion. Genetics.

[58] Ganetzky B. (1977). On the Components of Segregation Distortion in Drosophila Melanogaster. Genetics.

[59] Larracuente A.M., Presgraves D.C. (2012). The Selfish *Segregation Distorter* Gene Complex of *Drosophila melanogaster*. Genetics.

[60] Sandler L., Golic K. (1985). Segregation Distortion in Drosophila. Trends Genet..

[61] Jaenike J. (2001). Sex Chromosome Meiotic Drive. Annu. Rev. Ecol. Syst..

[62] Galizi R., Doyle L.A., Menichelli M., Bernardini F., Deredec A., Burt A., Stoddard B.L., Windbichler N., Crisanti A. (2014). A Synthetic Sex Ratio Distortion System for the Control of the Human Malaria Mosquito. Nat. Commun..

[63] Yu X., Zhao Z., Zheng X., Zhou J., Kong W., Wang P., Bai W., Zheng H., Zhang H., Li J. (2018). A Selfish Genetic Element Confers Non-Mendelian Inheritance in Rice. Science.

[64] Wang C., Wang J., Lu J., Xiong Y., Zhao Z., Yu X., Zheng X., Li J., Lin Q., Ren Y. (2023). A Natural Gene Drive System Confers Reproductive Isolation in Rice. Cell.

[65] Belfort M., Roberts R.J. (1997). Homing Endonucleases: Keeping the House in Order. Nucleic Acids Res..

[66] Stoddard B.L. (2011). Homing Endonucleases: From Microbial Genetic Invaders to Reagents for Targeted DNA Modification. Structure.

[67] Chevalier B.S., Stoddard B.L. (2001). Homing Endonucleases: Structural and Functional Insight into the Catalysts of Intron/Intein Mobility. Nucleic Acids Res..

[68] Windbichler N., Menichelli M., Papathanos P.A., Thyme S.B., Li H., Ulge U.Y., Hovde B.T., Baker D., Monnat R.J., Burt A. (2011). A Synthetic Homing Endonuclease-Based Gene Drive System in the Human Malaria Mosquito. Nature.

[69] Gogarten J.P., Senejani A.G., Zhaxybayeva O., Olendzenski L., Hilario E. (2002). Inteins: Structure, Function, and Evolution. Annu. Rev. Microbiol..

[70] Takeuchi R., Lambert A.R., Mak A.N.-S., Jacoby K., Dickson R.J., Gloor G.B., Scharenberg A.M., Edgell D.R., Stoddard B.L. (2011). Tapping Natural Reservoirs of Homing Endonucleases for Targeted Gene Modification. Proc. Natl. Acad. Sci. USA.

[71] Seligman L.M., Chisholm K.M., Chevalier B.S., Chadsey M.S., Edwards S.T., Savage J.H., Veillet A.L. (2002). Mutations Altering the Cleavage Specificity of a Homing Endonuclease. Nucleic Acids Res..

[72] Taylor G.K., Petrucci L.H., Lambert A.R., Baxter S.K., Jarjour J., Stoddard B.L. (2012). LAHEDES: The LAGLIDADG Homing Endonuclease Database and Engineering Server. Nucleic Acids Res..

[73] Wiedenheft B., Sternberg S.H., Doudna J.A. (2012). RNA-Guided Genetic Silencing Systems in Bacteria and Archaea. Nature.

[74] Gaj T., Gersbach C.A., Barbas C.F. (2013). ZFN, TALEN, and CRISPR/Cas-Based Methods for Genome Engineering. Trends Biotechnol..

[75] Mohanraju P., Makarova K.S., Zetsche B., Zhang F., Koonin E.V., van der Oost J. (2016). Diverse Evolutionary Roots and Mechanistic Variations of the CRISPR-Cas Systems. Science.

[76] Schiml S., Puchta H. (2016). Revolutionizing Plant Biology: Multiple Ways of Genome Engineering by CRISPR/Cas. Plant Methods.

[77] Hille F., Charpentier E. (2016). CRISPR-Cas: Biology, Mechanisms and Relevance. Philos. Trans. R. Soc. B Biol. Sci..

[78] Shabbir M.A.B., Shabbir M.Z., Wu Q., Mahmood S., Sajid A., Maan M.K., Ahmed S., Naveed U., Hao H., Yuan Z. (2019). CRISPR-Cas System: Biological Function in Microbes and Its Use to Treat Antimicrobial Resistant Pathogens. Ann. Clin. Microbiol. Antimicrob..

[79] Jiang F., Doudna J.A. (2017). CRISPR—Cas9 Structures and Mechanisms. Annu. Rev. Biophys..

[80] McFarlane G.R., Whitelaw C.B.A., Lillico S.G. (2018). CRISPR-Based Gene Drives for Pest Control. Trends Biotechnol..

[81] Noble C., Olejarz J., Esvelt K.M., Church G.M., Nowak M.A. (2017). Evolutionary Dynamics of CRISPR Gene Drives. Sci. Adv..

[82] Gantz V.M., Bier E. (2016). The Dawn of Active Genetics. Bioessays.

[83] Gantz V.M., Bier E. (2022). Active Genetics Comes Alive: Exploring the Broad Applications of CRISPR-based Selfish Genetic Elements (or Gene-drives). BioEssays.

[84] López Del Amo V., Bishop A.L., Sánchez C.H.M., Bennett J.B., Feng X., Marshall J.M., Bier E., Gantz V.M. (2020). A Transcomplementing Gene Drive Provides a Flexible Platform for Laboratory Investigation and Potential Field Deployment. Nat. Commun..

[85] Gantz V.M., Jasinskiene N., Tatarenkova O., Fazekas A., Macias V.M., Bier E., James A.A. (2015). Highly efficient Cas9-mediated gene drive for population modification of the malaria vector mosquito *Anopheles stephensi*. Proc. Natl. Acad. Sci. USA.

[86] Li M., Yang T., Kandul N.P., Bui M., Gamez S., Raban R., Bennett J., Sánchez C.H.M., Lanzaro G.C., Schmidt H. (2020). Development of a Confinable Gene Drive System in the Human Disease Vector Aedes Aegypti. eLife.

[87] Galizi R., Hammond A., Kyrou K., Taxiarchi C., Bernardini F., O’Loughlin S.M., Papathanos P.-A., Nolan T., Windbichler N., Crisanti A. (2016). A CRISPR-Cas9 Sex-Ratio Distortion System for Genetic Control. Sci. Rep..

[88] Champer J., Kim I., Champer S.E., Clark A.G., Messer P.W. (2019). Performance Analysis of Novel Toxin-Antidote CRISPR Gene Drive Systems. bioRxiv.

[89] Unckless R.L., Messer P.W., Connallon T., Clark A.G. (2015). Modeling the Manipulation of Natural Populations by the Mutagenic Chain Reaction. Genetics.

[90] Owen M. (2016). Diverse Approaches to Herbicide-Resistant Weed Management. Weed Sci..

[91] Bhowmik P.C. (1997). Weed Biology: Importance to Weed Management. Weed Sci..

[92] Verma P., Reeves R.G., Simon S., Otto M., Gokhale C.S. (2022). The Effect of Mating Complexity on Gene Drive Dynamics. Am. Nat..

[93] Moro D., Byrne M., Kennedy M., Campbell S., Tizard M. (2018). Identifying Knowledge Gaps for Gene Drive Research to Control Invasive Animal Species: The next CRISPR Step. Glob. Ecol. Conserv..

[94] Costea M., Weaver S.E., Tardif F.J. (2005). The Biology of Invasive Alien Plants in Canada. 3. *Amaranthus tuberculatus* (Moq.) Sauer Var. Rudis (Sauer) Costea & Tardif. Can. J. Plant Sci..

[95] Friesen L.F., Beckie H.J., Warwick S.I., Van Acker R.C. (2009). The Biology of Canadian Weeds. 138. *Kochia scoparia* (L.) Schrad. Can. J. Plant Sci..

[96] Keeley P.E., Carter C.H., Thullen R.J. (1987). Influence of Planting Date on Growth of Palmer Amaranth (*Amaranthus palmeri*). Weed Sci..

[97] Montgomery J.S., Sadeque A., Giacomini D.A., Brown P.J., Tranel P.J. (2019). Sex-Specific Markers for Waterhemp (*Amaranthus Tuberculatus*) and Palmer Amaranth (*Amaranthus Palmeri*). Weed Sci..

[98] Montgomery J.S., Giacomini D.A., Weigel D., Tranel P.J. (2021). Male-Specific Y-Chromosomal Regions in Waterhemp (*Amaranthus tuberculatus*) and Palmer Amaranth (*Amaranthus palmeri*). New Phytol..

[99] Rode N.O., Estoup A., Bourguet D., Courtier-Orgogozo V., Débarre F. (2019). Population Management Using Gene Drive: Molecular Design, Models of Spread Dynamics and Assessment of Ecological Risks. Conserv. Genet..

[100] Cavers P.B. (1983). Seed Demography. Can. J. Bot..

[101] Mithila J.G.A. (2013). Understanding Genetics of Herbicide Resistance in Weeds: Implications for Weed Management. Adv. Crop Sci. Technol..

[102] Gaines T.A., Duke S.O., Morran S., Rigon C.A.G., Tranel P.J., Küpper A., Dayan F.E. (2020). Mechanisms of Evolved Herbicide Resistance. J. Biol. Chem..

[103] Hirschberg J., McIntosh L. (1983). Molecular Basis of Herbicide Resistance in Plants in *Amaranthus hybridus*. Science.

[104] Gronwald J.W., Powles S.B., Holtum J.A.M. (1994). Resistance to Photosystem II Inhibiting Herbicides. Herbicide Resistance in Plants: Biology and Biochemistry.

[105] Cui Y., Xu J., Cheng M., Liao X., Peng S. (2018). Review of CRISPR/Cas9 SgRNA Design Tools. Interdiscip. Sci. Comput. Life Sci..

[106] Unckless R.L., Clark A.G., Messer P.W. (2017). Evolution of Resistance against CRISPR/Cas9 Gene Drive. Genetics.

[107] Noble C., Min J., Olejarz J., Buchthal J., Chavez A., Smidler A.L., DeBenedictis E.A., Church G.M., Nowak M.A., Esvelt K.M. (2019). Daisy-Chain Gene Drives for the Alteration of Local Populations. Proc. Natl. Acad. Sci. USA.

[108] Backus G.A., Delborne J.A. (2019). Threshold-Dependent Gene Drives in the Wild: Spread, Controllability, and Ecological Uncertainty. Bioscience.

[109] Powles S.B., Yu Q. (2010). Evolution in Action: Plants Resistant to Herbicides. Annu. Rev. Plant Biol..

[110] Kreiner J.M., Stinchcombe J.R., Wright S.I. (2018). Population Genomics of Herbicide Resistance: Adaptation via Evolutionary Rescue. Annu. Rev. Plant Biol..

[111] Svitashev S., Young J.K., Schwartz C., Gao H., Falco S.C., Cigan A.M. (2015). Targeted Mutagenesis, Precise Gene Editing, and Site-Specific Gene Insertion in Maize Using Cas9 and Guide RNA. Plant Physiol..

[112] Butt H., Rao G.S., Sedeek K., Aman R., Kamel R., Mahfouz M. (2020). Engineering Herbicide Resistance via Prime Editing in Rice. Plant Biotechnol. J..

[113] Wu J., Chen C., Xian G., Liu D., Lin L., Yin S., Sun Q., Fang Y., Wang Y. (2020). Engineering Herbicide-Resistant Oilseed Rape by CRISPR/Cas9-Mediated Cytosine Base-Editing. Plant Biotechnol. J..

[114] Sony S.K., Kaul T., Motelb K.F.A., Thangaraj A., Bharti J., Kaul R., Verma R., Nehra M. (2023). CRISPR/Cas9-mediated Homology Donor Repair Base Editing Confers Glyphosate Resistance to Rice (*Oryza sativa* L.). Front. Plant Sci..

[115] Wei T., Jiang L., You X., Ma P., Xi Z., Wang N.N. (2023). Generation of Herbicide-Resistant Soybean by Base Editing. Biology.

[116] Wu Y., Xiao N., Cai Y., Yang Q., Yu L., Chen Z., Shi W., Liu J., Pan C., Li Y. (2023). CRISPR-Cas9-Mediated Editing of the OsHPPD 3′ UTR Confers Enhanced Resistance to HPPD-Inhibiting Herbicides in Rice. Plant Commun..

[117] Shimatani Z., Kashojiya S., Takayama M., Terada R., Arazoe T., Ishii H., Teramura H., Yamamoto T., Komatsu H., Miura K. (2017). Targeted Base Editing in Rice and Tomato Using a CRISPR-Cas9 Cytidine Deaminase Fusion. Nat. Biotechnol..

[118] Green J.M., Owen M.D.K. (2011). Herbicide-Resistant Crops: Utilities and Limitations for Herbicide-Resistant Weed Management. J. Agric. Food Chem..

[119] Champer J., Reeves R., Yeon Oh S., Liu C., Liu J., Clark A.G., Messer P.W. (2017). Novel CRISPR/Cas9 Gene Drive Constructs in Drosophila Reveal Insights into Mechanisms of Resistance Allele Formation and Drive Efficiency in Genetically Diverse Populations. bioRxiv.

[120] Gantz V.M., Bier E. (2015). The Mutagenic Chain Reaction: A Method for Converting Heterozygous to Homozygous Mutations. Science.

[121] Bier E. (2022). Gene Drives Gaining Speed. Nat. Rev. Genet..

[122] Courtier-Orgogozo V., Danchin A., Gouyon P., Boëte C. (2020). Evaluating the Probability of CRISPR-based Gene Drive Contaminating Another Species. Evol. Appl..

[123] Crow J.F. (1991). Why Is Mendelian Segregation so Exact?. BioEssays.

[124] Simon S., Otto M., Engelhard M. (2018). Synthetic Gene Drive: Between Continuity and Novelty: Crucial Differences between Gene Drive and Genetically Modified Organisms Require an Adapted Risk Assessment for Their Use. EMBO Rep..

[125] James A.A. (2005). Gene Drive Systems in Mosquitoes: Rules of the Road. Trends Parasitol..

[126] Leung S., Windbichler N., Wenger E.A., Bever C.A., Selvaraj P. (2022). Population Replacement Gene Drive Characteristics for Malaria Elimination in a Range of Seasonal Transmission Settings: A Modelling Study. Malar. J..

[127] Kormos A., Lanzaro G.C., Bier E., Dimopoulos G., Marshall J.M., Pinto J., Dos Santos A.A., Bacar A., Rompão H.S.P.S., James A.A. (2021). Application of the Relationship-Based Model to Engagement for Field Trials of Genetically Engineered Malaria Vectors. Am. J. Trop. Med. Hyg..

[128] Annas G.J., Beisel C.L., Clement K., Crisanti A., Francis S., Galardini M., Galizi R., Grünewald J., Immobile G., Khalil A.S. (2021). A Code of Ethics for Gene Drive Research. Cris. J..

[129] Menz J., Modrzejewski D., Hartung F., Wilhelm R., Sprink T. (2020). Genome Edited Crops Touch the Market: A View on the Global Development and Regulatory Environment. Front. Plant Sci..

